# Peer review for medical journals: why and how?

**DOI:** 10.62675/2965-2774.20250098

**Published:** 2025-06-16

**Authors:** Wagner Luis Nedel, David Garcia Nora, Roberta Muriel Longo Roepke, Lívia Maria Garcia Melro, Márcio Manozzo Boniatti

**Affiliations:** 1 Hospital de Clínicas de Porto Alegre Department of Critical Care Porto Alegre RS Brazil Department of Critical Care, Hospital de Clínicas de Porto Alegre – Porto Alegre (RS), Brazil.; 2 Hospital de São Francisco Xavier Centro Hospitalar de Lisboa Ocidental Polyvalent Intensive Care Unit Lisbon Portugal Polyvalent Intensive Care Unit, Hospital de São Francisco Xavier, Centro Hospitalar de Lisboa Ocidental - Lisbon, Portugal.; 3 Universidade de São Paulo Faculdade de Medicina Hospital das Clínicas São Paulo SP Brazil Trauma and Acute Care Surgery Intensive Care Unit, Hospital das Clínicas, Faculdade de Medicina, Universidade de São Paulo - São Paulo (SP), Brazil.; 4 Hospital Samaritano Paulista Intensive Care Unit São Paulo SP Brazil Intensive Care Unit, Hospital Samaritano Paulista - São Paulo (SP), Brazil.

## WHY IS PEER REVIEW SO IMPORTANT?

Peer review is fundamental to scientific knowledge construction, ensuring the quality and reliability of research in an era of rapid information dissemination and limited content oversight. This became particularly evident during the COVID-19 pandemic, when the accelerated publication of preprints on platforms such as medRxiv and bioRxiv, while facilitating timely access to emerging data, also contributed to the spread of misinformation and public confusion—a phenomenon referred to as an "infodemic."

Being part of the peer review process is both a privilege and a responsibility. Researchers are motivated to contribute by not only academic altruism but also the desire to remain informed about emerging knowledge, critically assess relevant topics, and expand their academic networks.^(1)^

## ETHICAL ASPECTS OF PEER REVIEW

Journals provide reviewers with specific guidelines, but adherence to ethical standards, such as those set by the Committee on Publication Ethics (COPE),^(2)^ is essential for maintaining integrity and fairness in peer review.

Regardless of the review model, evaluations must be objective, impartial, and based solely on a manuscript's scientific quality. Reviewers should have verifiable expertise in the subject matter and disclose any potential conflicts of interest—personal, financial, professional, intellectual, political, or religious—before accepting a review assignment.^(2–4)^

Reviewers handle unpublished, confidential data, which must not be shared or discussed. If assistance from another expert is needed, journal approval must be obtained to ensure that confidentiality is maintained.^(2,4)^ Reviewers should also avoid uploading the manuscript to online platforms (e.g., Google Drive, Notion), as this may constitute a breach of confidentiality.

Reviewers must remain alert to signs of research misconduct, such as plagiarism, data fabrication, or image manipulation, and report any suspicions to the editor. As a reviewer, you should avoid using generative artificial intelligence tools to analyze or summarize manuscripts under review, unless explicitly permitted by the journal. The use of such tools must never compromise confidentiality or introduce bias.

## WHAT TO DO (AND WHAT NOT TO!) AS A PEER REVIEWER

### 1. Know the guidelines

**Figure 1:**
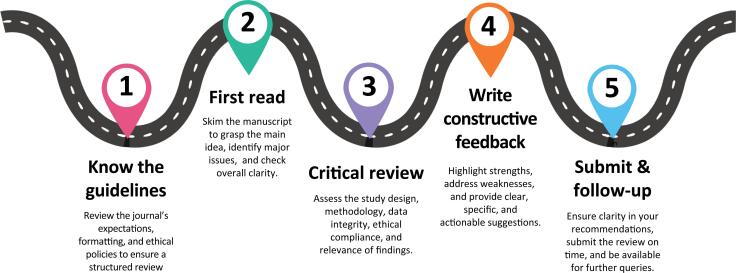
Peer review in practice: a process map with practical pointers.

Accept review requests only if you have the time and expertise. Journals are increasingly concerned with delays, and "time to first decision" is a key metric for authors. If you are facing a busy period or if the manuscript is outside your field, it is best to decline. An unfamiliar topic may lead to a vague review or important omissions (Figure 1).

### 2. First read

After accepting a review request, begin with an overview reading before a detailed assessment.^(5,6)^ If major methodological flaws compromise validity and rejection is likely, a concise review highlighting key issues is sufficient and respects both your time and the authors’.

### 3. Critical review

Focus your evaluation on the methodological soundness and scientific contribution of the manuscript, rather than making stylistic suggestions. Use structured tools, reporting guidelines (e.g., CONSORT, PRISMA), and resources such as the EQUATOR Network are used to guide your review. To support a thorough and standardized evaluation, reviewers are encouraged to consult reporting guidelines appropriate for the study design—such as CONSORT for randomized trials, PRISMA for systematic reviews, and STROBE for observational studies—available through the EQUATOR Network (www.equator-network.org).

### 4. Write constructive feedback

Provide clear, specific recommendations, referencing the relevant manuscript sections. Focus on methodology and scientific reasoning rather than writing preferences, particularly in high-quality manuscripts where minor flaws stand out. Be mindful of unconscious biases—comments should address the work, not the authors’ identity, affiliation, language, or background.^(7,8)^ Use a constructive, respectful tone. Additionally, remember that peer review connects people from diverse cultural and linguistic backgrounds. To foster inclusiveness, use clear and straightforward language and avoid idioms or jargon that may not translate well across contexts.

### 5. Submit and follow up

When submitting your review, ensure that your comments follow the journal's format and are well organized. After submission, respond to any follow-up questions promptly and be open to reviewing a revised version if invited. Respecting timelines and maintaining communication reinforce your professionalism and support a smooth editorial process.^(9)^ After completing a review, consider registering it on recognition platforms such as Web of Science Reviewer Recognition (formerly Publons) and linking it to your ORCID profile when permitted by the journal. These initiatives help formally acknowledge peer review as a meaningful academic contribution.

## WHAT DEFINES GOOD PEER REVIEW? SOME USEFUL TIPS

A reviewer must fulfill five key roles in the scientific publication process: a) evaluating the validity of the data and methodology; b) assessing whether conclusions align with results; c) providing constructive suggestions for improvement; d) determining the novelty, significance, and impact of findings; and e) ensuring an impartial, unbiased review.^(10,11)^

Organizing feedback systematically—by section or paragraph—improves clarity. A helpful structure begins with a brief introductory paragraph summarizing the manuscript's main findings and strengths. Next, comments should be clearly divided into major and minor points.^(12)^ Major comments refer to issues that affect the scientific validity, reproducibility, or ethical integrity of the study—for example, methodological flaws, missing ethical approval, incomplete statistical analysis, or unclear research questions. Minor comments may include typos, inconsistent formatting, unclear wording, or noncritical improvements in the presentation of results.

Finally, comments directed to authors must be distinguished from those intended for editors. "Comments to the authors" should focus on the research evaluation, providing detailed feedback for manuscript improvement. "Comments to the editor" should include justifications for the recommended publication decision, such as the study's relevance to the journal's audience, its scientific merit, and any major concerns (e.g., readability issues or flaws in study design).

Peer review is essential for ensuring the quality, validity, and reliability of scientific research. By providing objective, ethical, and constructive evaluations, reviewers help strengthen methodological standards and scientific integrity. This collaborative process not only enhances individual studies but also contributes to the responsible dissemination of knowledge. As peer review is a skill developed through practice and guidance, mentorship—especially for early-career researchers—can play a crucial role in building confidence and competence. Supporting such initiatives helps foster a new generation of qualified and responsible reviewers.
